# 

**Published:** 1893-02

**Authors:** 


### CONTENTS.

ORIGINAL COMMUNICATIONS—

A Case of Cretinism. By Hugh Hagan, M. D., Atlanta, Ga. .......... 705
The Therapeutics of Asiatic Cholera. By I. E. Atkinson, M. D. .......... 708
Some Points Obtained by a Three Months Sojourn in New York City. By W. C. Day, M. D., Winchester, Ill. .......... 715
The Relative Safety of Ether and Chloroform. By W. Roger Williams, F. R. C. S. .......... 723

SOCIETY REPORT—

The Clinical Society of Maryland .......... 730

CORRESPONDENCE—

Success in Plastic Surgery .......... 738

EDITORIAL—

The Medical Examiners Bill .......... 744
Vivisection .......... 746
The Amick Cure .......... 748
The Atlanta Polyclinic .......... 749

SELECTIONS .......... 750
MEDICAL ITEMS .......... 764
BOOK REVIEWS .......... 767
INDEX TO ADVERTISEMENTS .......... xii
ITEMS OF INTEREST .......... x-xi

